# Automated Magnetic Resonance Image Segmentation of Spinal Structures at the L4‐5 Level with Deep Learning: 3D Reconstruction of Lumbar Intervertebral Foramen

**DOI:** 10.1111/os.13431

**Published:** 2022-08-18

**Authors:** Tao Chen, Zhi‐hai Su, Zheng Liu, Min Wang, Zhi‐fei Cui, Lei Zhao, Lian‐jun Yang, Wei‐cong Zhang, Xiang Liu, Jin Liu, Shu‐yuan Tan, Shao‐lin Li, Qian‐jin Feng, Shu‐mao Pang, Hai Lu

**Affiliations:** ^1^ Department of Spinal Surgery Fifth Affiliated Hospital of Sun Yat‐sen University Zhuhai China; ^2^ School of Biomedical Engineering, Southern Medical University Guangdong Provincial Key Laboratory of Medical Image Processing Guangzhou China; ^3^ Department of Radiology Fifth Affiliated Hospital of Sun Yat‐sen University Zhuhai China; ^4^ School of Biomedical Engineering, Sun Yat‐sen University Shenzhen China; ^5^ School of Biomedical Engineering, Guangzhou Medical University Guangzhou China

**Keywords:** 3D reconstruction, Automated magnetic resonance image segmentation, Deep learning, Lumbar intervertebral foramen

## Abstract

**Objective:**

3D reconstruction of lumbar intervertebral foramen (LIVF) has been beneficial in evaluating surgical trajectory. Still, the current methods of reconstructing the 3D LIVF model are mainly based on manual segmentation, which is laborious and time‐consuming. This study aims to explore the feasibility of automatically segmenting lumbar spinal structures and increasing the speed and accuracy of 3D lumbar intervertebral foramen (LIVF) reconstruction on magnetic resonance image (MRI) at the L4‐5 level.

**Methods:**

A total of 100 participants (mean age: 42.2 ± 14.0 years; 52 males and 48 females; mean body mass index, 22.7 ± 3.2 kg/m^2^), were enrolled in this prospective study between March and July 2020. All participants were scanned on L4‐5 level with a 3T MR unit using 3D T2‐weighted sampling perfection with application‐optimized contrast with various flip‐angle evolutions (SPACE) sequences. The lumbar spine's vertebra bone structures (VBS) and intervertebral discs (IVD) were manually segmented by skilled surgeons according to their anatomical outlines from MRI. Then all manual segmentation were saved and used for training. An automated segmentation method based on a 3D U‐shaped architecture network (3D‐UNet) was introduced for the automated segmentation of lumbar spinal structures. A number of quantitative metrics, including dice similarity coefficient (DSC), precision, and recall, were used to evaluate the performance of the automated segmentation method on MRI. Wilcoxon signed‐rank test was applied to compare morphometric parameters, including foraminal area, height and width of 3D LIVF models between automatic and manual segmentation. The intra‐class correlation coefficient was used to assess the test‐retest reliability and inter‐observer reliability of multiple measurements for these morphometric parameters of 3D LIVF models.

**Results:**

The automatic segmentation performance of all spinal structures (VBS and IVD) was found to be 0.918 (healthy levels: 0.922; unhealthy levels: 0.916) for the mean DSC, 0.922 (healthy levels: 0.927; unhealthy levels: 0.920) for the mean precision, and 0.917 (healthy levels: 0.918; unhealthy levels: 0.917) for the mean recall in the test dataset. It took approximately 2.5 s to achieve each automated segmentation, far less than the 240 min for manual segmentation. Furthermore, no significant differences were observed in the foraminal area, height and width of the 3D LIVF models between manual and automatic segmentation images (*P* > 0.05).

**Conclusion:**

A method of automated MRI segmentation based on deep learning algorithms was capable of rapidly generating accurate segmentation of spinal structures and can be used to construct 3D LIVF models from MRI at the L4‐5 level.

## Graphical abstract

This study develops a method to complete the 3D reconstruction of lumbar intervertebral foramen at the L4‐5 level based on automated magnetic resonance image segmentation. Our approach can rapidly and accurately reconstruct the 3D lumbar intervertebral foramen model at the L4‐5 level, providing morphological data concerning anatomy, and surgical trajectory.

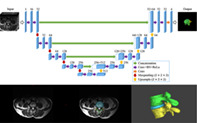

## Introduction

The lumbar intervertebral foramen (LIVF) approach is a common surgical approach of percutaneous endoscopic discectomy (PED) for treating lumbar disc herniation and spinal stenosis.^1^, ^2^ Establishing an appropriate working channel as well as planning an optimal trajectory are prerequisites to the success of PED.^3^ The optimal trajectory is critical to the successful entry of the obturator, endoscope, and other relevant instruments into the lumbar intervertebral foramen.^4^ In contrast, improper orientation can result in more fluoroscopy frequency, longer operation times, and incomplete views, increasing the possibility of complications.^5^, ^6^ With a narrow surgical field and a high reliance on the surgeon's skills, this LIVF approach of PED has a steep learning curve.^6^, ^7^

To avoid complications and decrease the learning curve, a thorough understanding of the anatomical structures of LIVF is crucial to mastering this procedure.^7^ It is well known that LIVF is a complex three‐dimensional (3D) anatomical structure.^8^, ^9^ The anatomical boundary of LIVF is composed of the upper and lower adjacent pedicles, the posterior and inferior margin of the upper vertebral body, the intervertebral disc, the posterior superior vertebral notch and the superior and inferior articular processes.^10^, ^11^ Most of the existing preoperative measuring methods involve 2D X‐ray images or MRI cross‐sections.^12^ However, these existing methods cannot precisely evaluate the relationship between the anatomical lumbar structure and the working channel, because the LIVF and trajectory are 3D. Free manipulation of the 3D LIVF model would help surgeons in evaluating the size of enlarging foramen and enable easy and safe trajectory planning for the LIVF approach of PED.^3^ But unfortunately, the current methods of reconstructing the 3D LIVF model are mainly based on manual segmentation, which is laborious and time‐consuming.

Recently, numerous interest‐based reports have addressed deep learning‐based approaches to automatic segmentation of spinal structures.^13^, ^14^, ^15^, ^16^ Previous studies have demonstrated the success of deep learning in the automatic MRI segmentation, but these studies have mainly focused on segmenting vertebra bodies and intervertebral discs without segmenting the vertebral notch and articular processes.^17^, ^18^ Few studies^19^, ^20^ have focused on the automatic segmentation of the vertebral notch and articular processes, essential to lumbar structures. Su *et al*.^20^ have developed a method for 3D reconstruction of Kambin's triangle, not for rapid 3D LIVF reconstruction. Liu *et al*.^19^ have developed a deep learning model for automatic MRI of vertebra bone structures (vertebra body, vertebral notch and articular processes) and intervertebral discs. But only lumbosacral intervertebral foramen was investigated in their work. Automated MRI segmentation of other lumbar levels was also required for rapid 3D LIVF reconstruction, especially at the L4‐5 level, which is the most frequently involved in lumbar spinal stenosis.^21^ To date, few have accomplished rapid 3D reconstruction of LIVF at the L4‐5 level, not to mention evaluating the size of foramen enlargement and planning the ideal trajectory of TF‐PED.

Therefore, it is urgent to develop an automated MRI segmentation method to reconstruct 3D LIVF model at the L4‐5 level accurately and rapidly. This study aims to assess: (i) whether deep learning is effective in achieving automated MRI segmentation of vertebra bone structures and intervertebral discs at the same time; and (ii) whether 3D reconstruction of LIVF at L4‐5 level based on automated MRI segmentation is rapid and reliable in clinical practice.

We hypothesized that automated MRI segmentation based on 3D‐UNet could be used to construct 3D LIVF models rapidly and accurately at the L4‐5 level.

## Methods

### Ethical Approval

This study was approved by the Ethics Committee of our hospital (2020 K05‐1), and the trial was registered online before initiation (NCT04647279). Forms of informed consent were signed by all participants before study began.

### Study Participants

A total of 100 L4‐5 levels in 100 participants (mean age: 42.2 ± 14.0 years; 52 males [52.0%] and 48 females [48.0%]; mean body mass index, 22.7 ± 3.2 kg/m^2^), were enrolled in the current study between March and July 2020. In the 100 L4‐5 levels, 29 levels were healthy and 71 levels were unhealthy, including three levels with spinal stenosis, 42 levels with disc herniation, and 26 levels with both. All participants in the study were scanned on L4‐5 level with a 3T MR unit (Magnetom Verio; Siemens, Erlangen, Germany) using 3D T2‐weighted sampling perfection with application‐optimized contrast with various flip‐angle evolutions (3D‐SPACE) sequences. 3D‐SPACE sequences enable high‐resolution multiplanar reformatted images and thin slice thicknesses to be used to examine small lesions and surrounding tissues.^22^, ^23^ The participants' demographics and MRI parameters of the datasets are outlined in Table 1.

**TABLE 1:** Dataset demographic breakdown

Sequence and Parameters	
3D T2‐SPACE^a^	
No. of participants^b^	
Men	52 (52)
Women	48 (48)
Age (years)	42.2 (22–82)
Men participants	40.7 (22–82)
Women participants	43.9 (23–74)
Body mass index (kg/m^2^)^c^	22.70 (22.07, 23.33)

*Notes*: Unless otherwise specified, data are means, with ranges in parentheses. 3D T2‐SPACE = 3D T2‐weighted sampling perfection with application‐optimized contrast with different flip‐angle evolutions.

^a^ 3D T2‐weighted sampling perfection with application‐optimized contrast with different flip‐angle evolutions sequence with the following parameters: repetition time msec/echo time msec, 2800.0/189.0; flip angle, 45°; field of view, 240 × 240 mm; matrix, 320 × 320; section thickness, 0.8 mm; bandwidth, 579 kHz; and final image resolution, 0.8 × 0.8 × 0.8 mm.

^b^ Data are numbers of patients, with percentages in parentheses.

^c^ Data are means, with 95% confidence intervals in parentheses.

### Inclusion and Exclusion Criteria

The inclusion criteria included the following PICOS principles: (i) participants aged > 18 years old; (ii) underwent lumbar spine MRI examination with 3D SPACE sequences in the supine position; (iii) VBS and IVD segmented form images; (iv) foraminal area, height, and width at the L4‐5 levels; and (v) a prospective study. The exclusion criteria of the participants in the current study included the following: (i) history of previous lumbar spinal surgery; (ii) anatomic abnormalities; (iii) lumbar spondylolisthesis; and (iv) severe artifacts of images.

### Image Annotation and Data Preprocessing

Manual segmentation was performed using Mimics 19.0 (Materialise, Leuven, Belgium) after all images were saved in DICOM format. The segmentation of VBS and IVD were performed manually by one surgeon with more than 5 years of experience, according to their anatomical outlines.

In addition, all segmented masks were assessed by an expert radiologist and a different expert surgeon. Any disagreements of segmentations were openly discussed and revised among the three doctors. The revised manual segmentations of the VBS and IVD were served as the ground truth. Lastly, all manual segmentation files were exported as NIfTI files for preprocessing.

Cropping, normalizing, and padding were all applied to all images whereby given an image $I\in {\mathbb{R}}^{D\times H\times W}$, the cropped image $I_{\text{crop}}$ was acquired as follows:

$$I_{\text{crop}}\left(I\right)=I\left[:,\frac{1}{4}H-10:\frac{3}{4}H+10,\frac{1}{4}W-20:\frac{3}{4}W\right],$$

*D*, *H*, and *W* are the depth, height, and width of the image. In this dataset, H=W=320 and *D* varied from 88 to 128. By subtracting the average voxel value from the standard deviation of the voxels, the cropped image size was D×180×180. Image normalization was eventually padded with 128×180×180 by padding normalized images with zeros.

### Deep‐learning Algorithm and Experimental Configurations

**Fig. 1:**
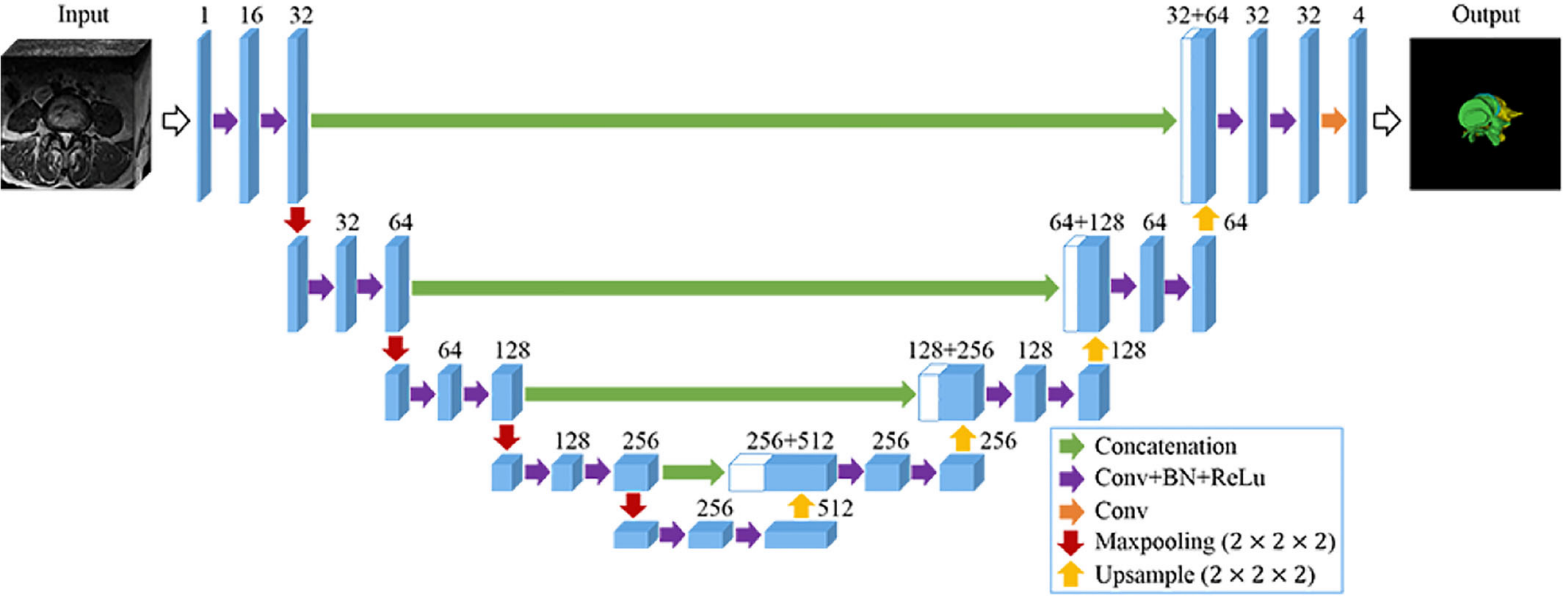
The 3D‐UNet architecture. The blue boxes denote feature maps. The number of channels is denoted above each feature map. The arrows of different colors indicate different operations

A deep‐learning algorithm based on 3D‐UNet^24^ was used in this study for automatic segmentation of lumbar spine structures. As shown in Fig. 1, the 3D‐UNet was composed of an encoder (the left path) and a decoder (the right path). The input of 3D‐UNet was a 128×180×180 MRI with a channel. In the output, there were four channels, one for background, one for upper vertebra, one for IVD, and one for lower vertebra. L4 and L5 vertebrae were represented by the upper and lower vertebrae at L4‐5 level. The size of convolutional kernels was 3×3×3 except the last convolutional layer, which used a 1×1×1 convolutional kernel. By implementing trilinear interpolation, the upsample module was implemented.

In this study, performance was evaluated using a five‐fold cross‐validation. Using a randomization method, each dataset was divided into five groups, each containing 20 samples. As training dataset for the automated segmentation model, four groups of 80 samples were used, while a group of 20 samples was used as the test dataset. A random sample of 10 samples was selected for validation during training. For each experiment, there was a total of 70 samples for training, 10 samples for validation, and 20 samples for testing. The 3D‐UNet was trained in Pytorch 1.5.1 (open‐source, Facebook Artificial Intelligence Research) using the Adam optimizer and a batch size of two epochs. A learning rate of 0.0005 was initially set, but subsequently lowered by five times at epochs 33 and 66. An RTX 2080Ti GPU (Nvidia, Santa Clara, CA, USA) was used for training and testing the 3D‐UNet for 2.5 s per subject, which lasted approximately 7 h.

### Performance Evaluation

The dice similarity coefficient (DSC), precision, and recall were applied as the quantitative metrics to assess the segmentation performance.^17^, ^25^ DSC measures the similarity between automatic and manual segmentation (TP = true positive, FP = false positive, FN = false negative). An accurate classification is determined by the recall, which is the proportion of true positives. The precision is the proportion of true positives among all positive classifications. The following are the equations of the three metrics:

$$\mathrm{DSC}=2\times \mathrm{TP}/\left(\mathrm{FP}+2\mathrm{TP}+\mathrm{FN}\right),$$

$$\text{Precison}=\mathrm{TP}/\left(\mathrm{TP}+\mathrm{FP}\right),$$

$$\text{Recall}=\mathrm{TP}/\left(\mathrm{TP}+\mathrm{FN}\right).$$

Scores (*x*) of 0 ≤ *x* < 0.7 would indicate poor performance, 0.7 ≤ *x* < 0.8 would indicate fair performance, 0.8 ≤ *x* < 0.9 would indicate good performance, and *x* ≥ 0.9 would indicate excellent performance.^26^ Metrics were calculated in the original image space for each object (VBS or IVD). The averages were then taken across all metrics for the five‐fold cross‐validation datasets.

### Morphometric Parameters

The performance of the automated segmentation method was evaluated, followed by the morphometry analysis of 3D LIVF models using manual and automatic segmentation images.

### Foraminal Area

**Fig. 2:**
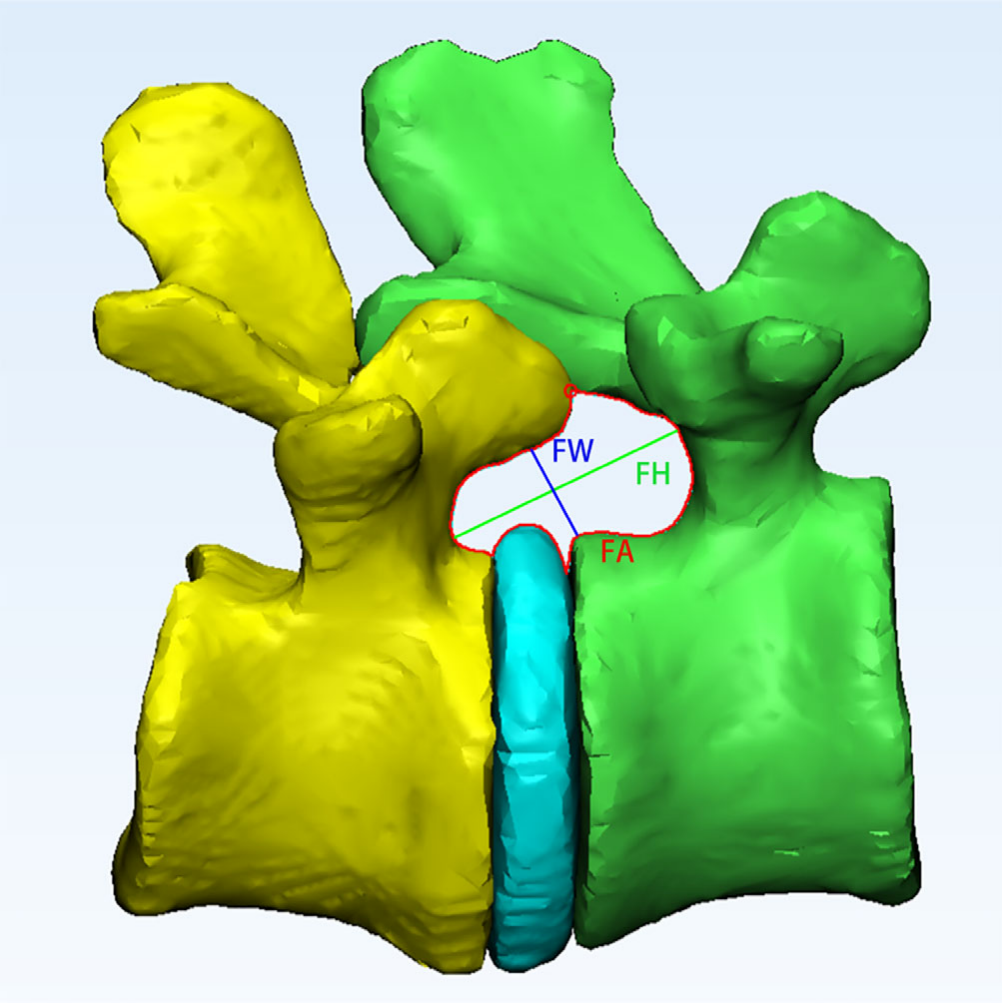
The LIVF dimensions were measured at the lateral views. The LIVF height (FH) was defined as the longest distance between the cranio‐caudal boundary (green line); the width (FW) was defined as the shortest distance between the postero‐inferior corner of the proximal vertebrae and the opposing boundary (blue line); and the area (FA) was drawn with the temporary boundaries set at 0.5 mm increments (red circle) according to the 3D LIVF model outline (red line)

An outline of the LIVF was used to define the foraminal area.^27^ It determined by measuring the boundary of the adjacent superior and inferior vertebral pedicles, the posterosuperior portion of the inferior vertebral body, the posterior portion of the intervertebral disc, the posteroinferior portion of the superior vertebral body and the anterior portion of the articular processes (Fig. 2).

### Foraminal Height

The height of the LIVF was defined as the longest distance between the boundary of the superior and inferior pedicle.^27^, ^28^, ^29^ As shown in Fig. 2, it was measured from the inferior aspect of the upper pedicle to the superior aspect of the lower pedicle.

### Foraminal Width

The width of the LIVF was defined as the shortest distance between the posteroinferior corner of the superior vertebrae and the opposing boundary.^28^ Figure 2 shows the measurement made on a line through the posteroinferior corner of the superior vertebra and vertical to the anterior surface of the opposing facet.

Three morphometric parameters were measured for the 3D LIVF models derived from manually or automatically segmented images, using 3‐Matic. First, three morphometric parameters, including the foraminal area, height, and width, were measured separately by two observers. One month later, all morphometric parameters of 3D LIVF models were re‐measured by one of the observers in this study. Calculations for the intraclass correlation coefficient were hence obtained to assess the test‐retest reliability and inter‐observer reliability of multiple measurements.

### Statistical Analysis

In this study, SPSS 26.0 (IBM Corporation, Chicago, IL, USA) was used to analyze all statistical tests. Without assuming the underlying distribution, Pearson's coefficients of correlation and Wilcoxon signed‐rank tests were used to compare the associated differences of these morphometric parameters between automatically and manually segmented 3D‐LIVF models. The significant difference was set at a *P*‐value <0.05. The intra‐class correlation coefficients (ICCs) were also used to assess the test‐retest reliability and inter‐observer reliability of multiple measurements for the described morphometric parameters of the 3D LIVF model. An intra‐class correlation coefficient of 0 to 0.20 indicates slight agreement; 0.21 to 0.40 indicates fair agreement; 0.41 to 0.60 indicates moderate agreement; 0.61 to 0.80 indicates substantial agreement; and 0.81 to 1 indicates almost perfect agreement.

## Results

### Performance and Speed

The segmentation performance of all spinal structures (VBS and IVD) was found to be 0.918 (healthy levels: 0.922; unhealthy levels: 0.916) for the mean DSC, 0.922 (healthy levels: 0.927; unhealthy levels: 0.920) for the mean precision, and 0.917 (healthy levels: 0.918; unhealthy levels: 0.917) for the mean recall in the test dataset. The detailed quantitative segmentation metrics evaluation was highlighted in Table 2 and Table 3.

**TABLE 2:** Results of automatic segmentation performances of the dataset

Performances and dataset	DSC			Precision			Recall		
	VBS	IVD	VBS and IVD	VBS	IVD	VBS and IVD	VBS	IVD	VBS and IVD
Training	0.942 (0.941, 0.943)	0.929 (0.927, 0.932)	0.938 (0.936, 0.939)	0.942 (0.941, 0.944)	0.932 (0.928, 0.937)	0.939 (0.937, 0.941)	0.942 (0.941, 0.943)	0.928 (0.924, 0.931)	0.937 (0.936, 0.939)
Validation	0.927 (0.924, 0.930)	0.908 (0.894, 0.921)	0.920 (0.914, 0.927)	0.929 (0.924, 0.934)	0.910 (0.890, 0.931)	0.923 (0.914, 0.931)	0.925 (0.920, 0.930)	0.909 (0.894, 0.923)	0.920 (0.913, 0.926)
Test	0.925 (0.919, 0.930)	0.904 (0.892, 0.916)	0.918 (0.910, 0.926)	0.927 (0.920, 0.934)	0.911 (0.895, 0.927)	0.922 (0.912, 0.932)	0.924 (0.919, 0.929)	0.904 (0.891, 0.918)	0.917 (0.910, 0.925)

Note: Data are means of 5‐Fold Cross‐validation scores, with 95% confidence intervals in parentheses.

Abbreviations: DSC, Dice similarity coefficient; IVD, intervertebral discs; VBS, vertebra bone structures.

**TABLE 3:** The mean accuracy results of all spinal structures at the test dataset

Performances and test dataset	Diagnosis			
	Healthy	SS	DH	SS and DH
DSC	0.922	0.932	0.914	0.918
Precision	0.927	0.944	0.914	0.925
Recall	0.918	0.920	0.918	0.915

Note: At 100 L4/5 levels, there were 29 healthy levels, 71 unhealthy levels with spinal stenosis: 3 levels, disc herniation: 42 levels, or both: 26 levels.

Abbreviations: DH, disc herniation; SS, spinal stenosis; SS and DH, spinal stenosis and disc herniation.

The entire training of the 3D‐UNet took about 7 h in each cross‐validation fold. After training, the 3D‐UNet was able to complete the automatic segmentation of each subject within 2.5 s. Therefore, this method took much less time than the 240 min required for manual segmentation.

### Morphometric Analysis of 3D LIVF Models

**Fig. 3:**
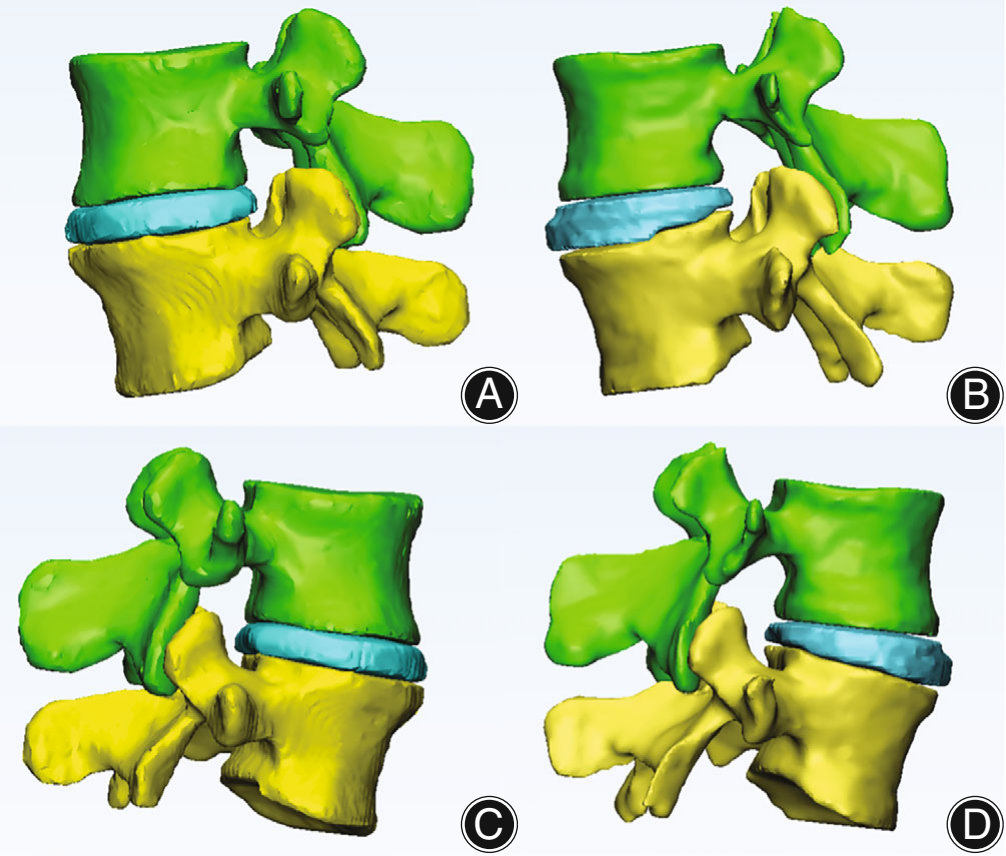
Example 3D LIVF models show comparison between automatic segmentation, (A–C), and manual segmentation, (B–D). (A), (B) in left views and (C), (D) in right views

Results showed that the method based on 3D‐UNet could successfully segmented spinal structures (VBS and IVD) on axial MRI. Furthermore, the 3D LIVF models were compared between the automatic segmentation (Fig. 3A,C) and the manual segmentation (Fig. 3B,D).

### Foraminal Area

**Fig. 4:**
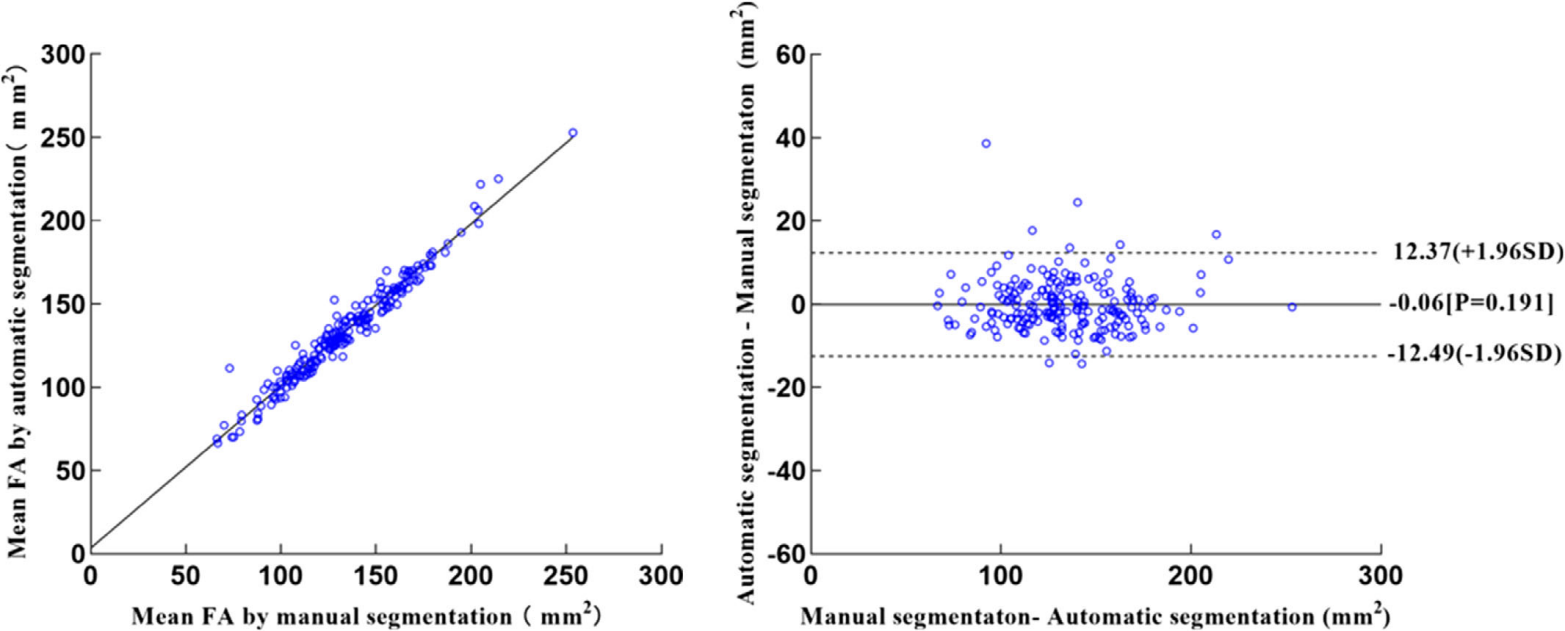
Scatterplots (left) and Bland–Altman plots (right) show comparison of FA meansurements of L4‐5 test dataset produced from manual and automatic segmentation methods. FA = foraminal area

No significant differences were observed in the foraminal area of the 3D LIVF models between manual and automatic segmentation images (*P* = 0.191). The mean area of 3D LIVF was between 133.40 ± 30.47 mm^2^ and 133.33 ± 30.32 mm^2^ on manual segmentation and automated segmentation (*R* = 0.978), respectively. The scatterplots and Bland‐Altman plots of the test dataset were as depicted in Fig. 4.

### Foraminal Height

**Fig. 5:**
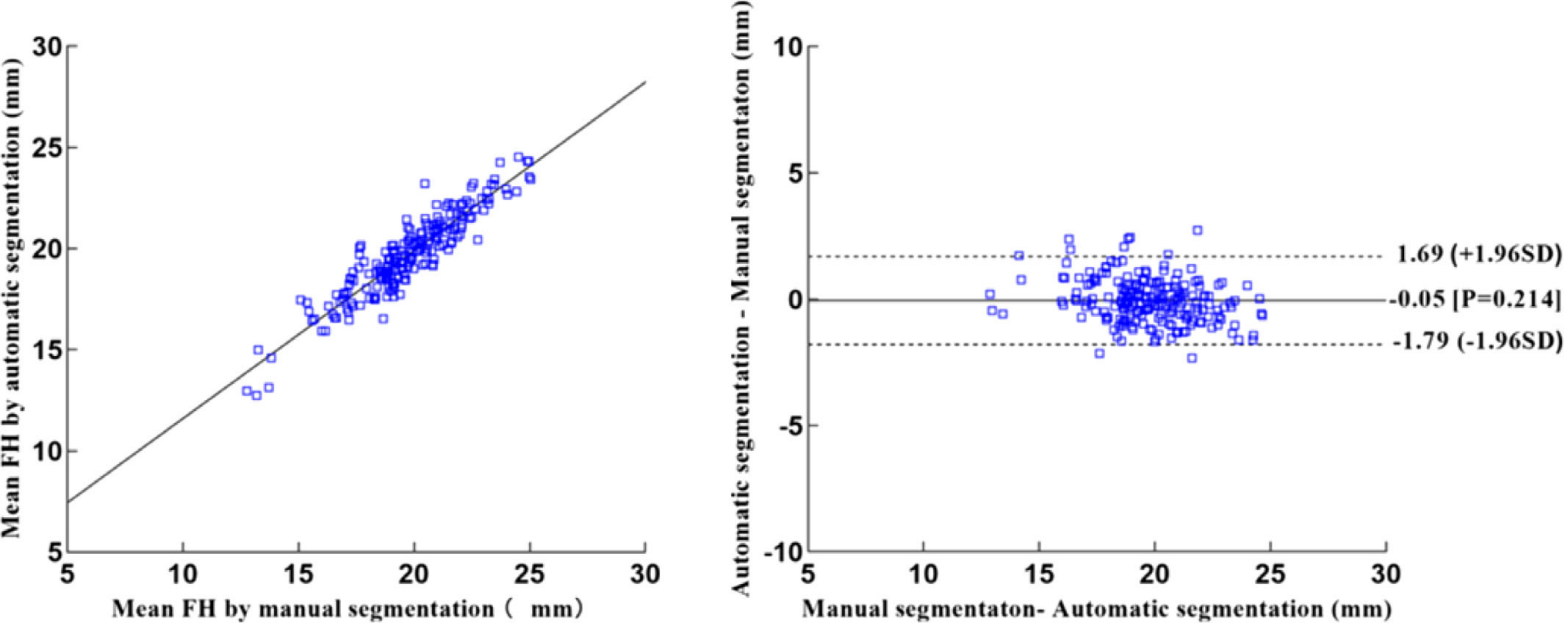
Scatterplots (left) and Bland–Altman plots (right) show comparison of FH meansurements of L4‐5 test dataset produced from manual and automatic segmentation methods. FH = foraminal height

No significant differences were observed in the foraminal height of the 3D LIVF models between manual and automatic segmentation images (*P* = 0.214). The mean height of 3D LIVF was between 19.75 ± 2.31 mm and 19.70 ± 2.08 mm on manual segmentation and automated segmentation (*R* = 0.924), respectively. Foraminal height was 15 millimeters or less in five of the 200 LIVF models generated from manual segmentation, similar to five of the 200 LIVF models generated from automatic segmentation. Figure 5 shows scatterplots and Bland‐Altman plots of the test dataset.

### Foraminal Width

**Fig. 6:**
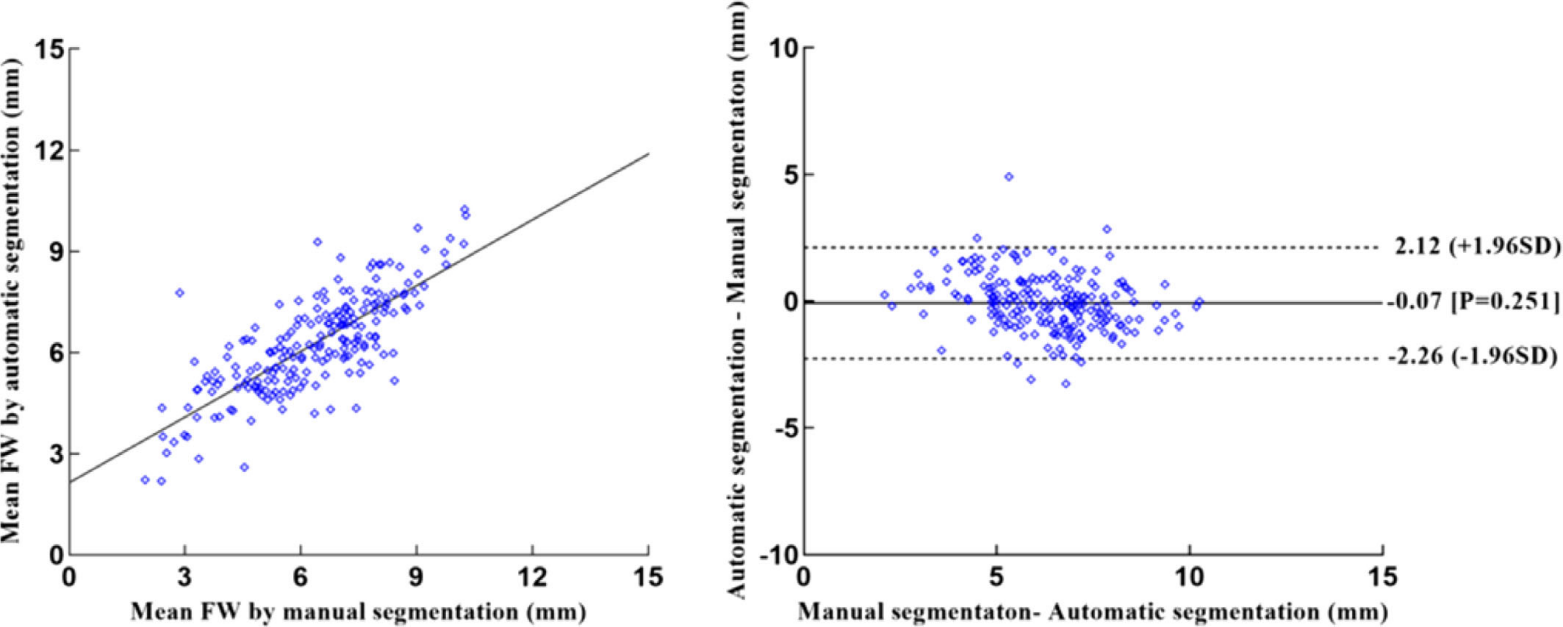
Scatterplots (left) and Bland–Altman plots (right) show comparison of FW meansurements of L4‐5 test dataset produced from manual and automatic segmentation methods. FW = foraminal width

No significant differences were observed in the foraminal width of the 3D LIVF models between manual and automatic segmentation images (*P* = 0.251). The mean height of 3D LIVF was between 6.30 ± 1.77 mm and 6.23 ± 1.48 mm on manual and automated segmentation (*R* = 0.778), respectively. Foraminal width was 8 millimeters or less in 169 of the 200 LIVF models generated from manual segmentation, which is less than 180 of the 200 LIVF models generated from automatic segmentation. Figure 6 shows scatterplots and Bland‐Altman plots of the test dataset.

### Reliability

The intra‐class correlation with 3D LIVF models from manual segmentation (automated segmentation) was found to be 0.994 (0.999) for the foraminal area, 0.991 (0.992) for the foraminal height and 0.992 (0.985) for the foraminal width. The interclass correlation with 3D LIVF models from manual segmentation (automated segmentation) was 0.991 (0.995) for the foraminal area, 0.983 (0.988) for the foraminal height, and 0.990 (0.984) for the foraminal width. There have been excellent ICCs in all measurements.

## Discussion

In this study, deep learning method was shown to perform well in segmenting lumbar spinal structures (VBS and IVD). Reconstructing the 3D LIVF model on MRI using this method can be done rapidly and accurately. Furthermore, morphometric parameters of 3D LIVF models were not significantly different among automatic and manual segmentation methods in this study. Moreover, the reliability test demonstrated a strong test‐retest reliability and inter‐observer reliability of morphometric measurements.

### Performance Analysis of Automated Segmentation

Deep learning‐based methods are popular in vertebrae segmentation of CT data^16^, ^30^ and IVD segmentation of MRI data.^14^, ^31^ Janssens *et al*.^16^ proposed a cascaded 3D fully convolutional network‐based method for the automated segmentation of lumbar vertebrae from CT data. Their methods have achieved a mean DSC of 0.958 for all lumbar vertebrae and 0.954 for L4 vertebrae, taking an average of 79 s to finish the segmentation of one CT data. For automated segmentation of IVD, Li *et al*.^14^ developed a 3D multi‐scale context fully convolutional network, which achieved a mean segmentation DSC of 0.912, taking an average of 9 s to segment IVD from MRI data. However, the exiting deep learning‐based works of the automated segmentation of spinal structures mainly include separate vertebrae and IVD, failed to consider the dependencies between the adjacent structures.^13^, ^14^ In our study, the deep learning‐based method achieved the simultaneous segmentation of vertebrae bone structures and IVD with a mean DSC of 0.918 for spinal structures (VBS and IVD), 0.925 for VBS and 0.904 for IVD. According to these results, our method has shown excellent performance when it comes to segmenting spinal structures from MRIs. In addition, after training, our deep learning‐based method could accomplish an automatic segmentation from MRI dates in 2.5 s, which is faster than previous studies. Notably, the segmentation performance of healthy levels was identical to unhealthy levels, because the performance of our method was not generally affected by lumbar diseases.

### Three‐dimensional Reconstruction of Lumbar Intervertebral Foramen

To the best of our knowledge, this study is the first to investigated 3D reconstruction of LIVF and generated 3D models at the L4‐5 level, using a deep learning‐based method. No significant differences were observed in the foraminal area, height and width of the 3D LIVF models between manual and automatic images. In addition, our results showed similar measurements of foraminal height from 3D models to the previous studies at the L4‐5 level.^28^, ^32^, ^33^ The mean of foraminal width from 3D models was consistent with Senoo et al.'s study.^29^ However, the results of the foraminal area from 3D LIVF models were smaller than the results in Zhong et al.'s study^28^ but were larger than Iwata et al.'s study.^27^ Different imaging modalities and measurement approaches may contribute to these differences. Moreover, 3D reconstruction from automated MRI segmentation of VBS and IVD can generate a complete and accurate 3D LIVF models, which is more advantageous for morphometric analysis.

### Clinical Application of 3D LIVF Models

A long learning curve is associated with PED because LIVF has a complex anatomical structure.^7^ In order to avoid complications and decrease the learning curve, it is crucial to be well versed in the anatomical structure of LIVF.^6^, ^7^ Automated segmentation using a deep learning algorithm leads to rapid generation of 3D LIVF models, which provides detailed and commonplace information of the surgical field. Free manipulation of the 3D model will quickly identify the ideal trajectory of PED, thereby helping junior surgeons to master the 3D anatomy LIVF and evaluate the size of foramen.

Surgeons may need to perform foraminoplasty to enlarge the LIVF during PED surgery, especially in patients with foraminal stenosis. To determine the need of a suitable foramen enlargement during the procedure mainly depends on the foraminal stenosis condition and whether the working channel size is satisfied. When the foraminal height was 15 millimeters or less,^34^ or the foraminal width was 8 millimeters or less,^35^ a suitable foramen enlargement for PED was required.

## Limitations

This study had some limitations. First, the scan time with MRI was considerably longer than CT. However, the imaging time of about 8 min for the detection of each lumbar level in MRI was acceptable. This pilot study only explored segmentation on the L4‐5 level. Other 3D reconstructions using automated magnetic resonance image segmentation for different spinal regions (thoracic and cervical) need to be studied in the future. To evaluate the clinical efficacy of the described method, further clinical experiments will be also required. Despite these limitations, the present study still demonstrates a promising performance of the deep learning method in the automatic segmentation of lumbar spinal structures (VBS and IVD) at the L4‐5 level.

## Conclusion

This present study increased the ability of automated VBS and IVD segmentation to speed, accuracy and precision. Automated MRI segmentation can segment spinal structures seemingly within the near‐human expert performance and demonstrate the efficacy in constructing 3D LIVF models at the L4‐5 level. The goal of this study is to provide a new method for reconstructing 3D LIVF models, which provides an important step toward surgical trajectory planning for PED at the L4‐5 level.

### Author Contribution

Conception and design of study: H. Lu, S‐M. Pang, Q‐J. Feng, and S‐L. Li; Acquisition of data: T. Chen, Z‐H. Su, Z. Liu, Min. Wang, and Z‐F. Cui; Analysis and interpretation of data: L. Zhao, L‐J. Yang, W‐C. Zhang, X. Liu, J. Liu, and S‐Y. Tan. All authors discussed the results and contributed to the final manuscript.

Tao Chen, Zhihai Su, and Zheng Liu equally contributed to this work as first authors. Shumao Pang and Hai Lu equally contributed to this work as corresponding authors.

## Availability of Data and Materials

The datasets generated and analyzed during the current study are not publicly available but may be available from the corresponding author on reasonable request.
